# Labeling Clinical Notes With Large Language Models: Retrospective Benchmarking Study

**DOI:** 10.2196/81708

**Published:** 2026-11-10

**Authors:** Pritam Mukherjee, Michael Nazmifar, Vivek Batheja, Ricardo Bigolin Lanfredi, Abhinav Suri, Anisa Prasad, Nicole Tran, Sydney Lewis, Nusrat Rabbee, Ronald M Summers

**Affiliations:** 1 Imaging Biomarkers and Computer Aided Diagnosis Lab, Radiology and Imaging Sciences, National Institutes of Health Clinical Center, 10 Center Dr, Building 10, Rm 1C224, Bethesda, MD, 20892, United States, 1 301-402-5486; 2 Biostatistics and Clinical Epidemiology Service, National Institutes of Health Clinical Center, Bethesda, MD, United States

**Keywords:** large language models, clinical notes, information extraction, benchmarking, health records

## Abstract

**Background:**

Clinical notes are rich sources of clinical information and are routinely used in clinical practice to infer past diagnoses and other comorbidities. Large language models (LLMs) may simplify the task by extracting this information from clinical notes.

**Objective:**

The study aims to evaluate the performance of state-of-the-art LLMs on the task of labeling English clinical notes.

**Methods:**

This institutional review board–approved retrospective study comprised 380 clinical notes obtained from 2 institutions in the United States. The notes were manually labeled for the presence or absence of 29 conditions (simplified Elixhauser Comorbidity Index) by 2 human readers—a radiology resident and a medical student under the supervision of a senior radiologist. Forty-six LLMs, including 4 closed-source commercial models from the GPT and Claude families, and 42 open-weights LLMs from the Llama (13 models) and the Qwen (29 models) families were used to label each note. The GPT-o3-mini-high reasoning model was used to parse the free-text model responses to binary yes/no answers. Using the manual labels as the reference standard, the recall, precision, and *F*_1_-scores for the models were evaluated. The effect of factors such as the length of the notes and the family, size, and release recency of the LLMs was analyzed using generalized linear models.

**Results:**

The top-performing LLMs included the GPT and Claude models, and the larger open-weights models included Llama 3 70B, Llama 3.1 70B, Llama 3.1 405B, Llama 3.3 70B, Qwen 1.5 72B, Qwen 1.5 110B, Qwen 2 72B, and Qwen 2 VL 72B, which achieved *F*_1_-scores above 70%. The most recent and higher-capacity releases of the LLMs have been improving mainly in their precision. The evaluation of 29 conditions showed a large variance in performance depending on the condition.

**Conclusions:**

In this evaluation with 46 LLMs, larger open-weights LLMs such as the Llama 3.3 70B performed on par with or better than the GPT and Claude models, achieving *F*_1_-scores above 70%. The performance of open-weights models improved significantly with model size and recency. These results suggest that locally run open-weights LLMs can be potentially used to label clinical notes.

## Introduction

Clinical notes are a crucial part of patient care, containing relevant medical data not captured elsewhere in the structured data (eg, diagnosis codes, laboratory test data, etc) of electronic medical records. The unstructured clinical narrative often provides comprehensive documentation of patients’ medical histories, diagnoses, treatments, and other critical information. Despite their importance, these notes are often lengthy, disorganized, and challenging to navigate. As a result, reviewing and analyzing large quantities of clinical notes can be a time-consuming and error-prone task. Additionally, the transition from paper records to electronic health records has inadvertently increased the volume of these notes since templates and copy-paste functions have introduced redundant information and unnecessary data into clinical notes [1,2]. This information overload further complicates the identification of critical clinical data and can potentially result in unfavorable outcomes for both patients and physicians.

Naturally, there has been a longstanding interest in developing natural language processing (NLP) tools to extract information from free-text clinical notes [3]. With the recent advent of large language models (LLMs), many studies have investigated the efficacy of using LLMs for this task [4-7]. In our paper, we analyze 46 LLMs, which is an order of magnitude more than prior studies, and evaluate their performance across a much broader range of abnormality types. This comprehensive analysis allows us to uncover trends related to model capacity and recency, revealing considerable variability in performance depending on the condition examined. Compared to older rule-based NLP techniques, deep-learning–based LLMs perform better and provide more flexibility by allowing prompting in natural language rather than writing bespoke rules for each different information extraction task [8-11]. Besides information extraction, these LLMs can potentially be used for many other clinically relevant tasks such as clinical decision support, answering medical questions, and generating medical notes and reports [12].

In this study, we focus on a prototypical information extraction task: the task of labeling clinical notes with the presence or absence of a set of clinical diagnoses. This task is useful in both clinical and research contexts. For example, in the clinical context, the presence or absence of comorbidities can, in part, aid in diagnosis, prognosis, and determining the appropriate course of treatment. While *International Classification of Diseases, Ninth/Tenth Revision (ICD-*9/*ICD*-10) codes in the structured electronic medical record can be potentially used for this purpose, it has been demonstrated that these codes, which are primarily for billing purposes, are often inconsistent across institutions [13], are prone to mistakes and omissions, particularly for secondary diagnoses and comorbidities [14], and may be unreliable in many contexts [15]. Additionally, in inpatient settings, the free-text progress notes may be the primary source of diagnosis information, as the assignment of billing codes often lags the course of treatment. In a research context, identifying patients with or without certain diagnoses is a typical step for curating patient cohorts via inclusion/exclusion criteria for clinical studies.

From a practical perspective, this task provides several advantages: first, different diagnoses may have to be inferred from the notes in various ways (including the presence of certain medications or lab test results), leading to a diversity in the test set. Second, the binary outcomes from the task are highly amenable to statistical analyses. A related task is summarizing notes; however, evaluating the free-text summarization requires careful manual review, which does not scale when a large number of notes (380 in our case) and models (46 in our case) are evaluated.

Prior work on this task has adopted many strategies, including rule-based NLP techniques, as well as traditional machine learning and deep learning models [16-19]. The possibility of using LLMs to label clinical notes has also been explored, and both the potential for higher performance and pitfalls related to hallucinations and inconsistent performance have been highlighted [20,21]. Here, our aim is to benchmark the performance of both closed-source commercial models, including GPT and Claude models, and 2 popular open-weights model families Llama and Qwen [22-27]. An advantage of the open-weights LLMs is that they can be used locally in a privacy-preserving manner, which can be an important consideration in the medical context. Additionally, we explore performance differences across different model families, model sizes, and model recency and the effect of the length of the notes on the performance.

## Methods

### Ethical Considerations

This was a retrospective study approved by the Institutional Review Board at the National Institutes of Health (NIH) Clinical Center (number 000037). Informed consent was waived. All patient notes used in this study were deidentified before use. Participants received no compensation because the study involved retrospective analysis of existing data without direct participant involvement.

### Data

In this retrospective study approved by the institutional review board, we used 2 datasets from 2 medical institutions, the NIH Clinical Center (center 1) in Bethesda, United States, and the Beth Israel Deaconess Medical Center (center 2) in Boston, United States. The first dataset (referred to as NIH, henceforth) comprised 180 history and physical examination notes corresponding to the first visits of 180 participants randomly chosen from patients who were admitted and underwent computed tomography scans at center 1 between 2000 and 2023. The second dataset (Medical Information Mart for Intensive Care [MIMIC]), a subset of the publicly available MIMIC-IV-Note dataset [28,29], consisted of 200 discharge notes corresponding to 200 participants randomly chosen from patients admitted to the hospital and emergency department at center 2. The notes were deidentified before use with the Stanford deidentifier tool and reviewed by authors MN and VB [30].

### Reference Standard Labeling of Free-Text Notes

Each note was labeled for the presence or absence of 29 conditions, a simplified version of the 30 conditions covered by the Elixhauser Comorbidity Index (ECI) [31]. The 2 comorbidities of “diabetes, uncomplicated” and “diabetes, complicated” in the ECI are merged to a single category called “diabetes” (the full list is available in Table 1). The ECI was used as a framework for selecting a broad and clinically diverse set of conditions that may be documented in admission and discharge notes and may be relevant targets for automated clinical information extraction. The annotation task was not intended to reproduce formal Elixhauser coding, calculate an ECI score, or assign billing codes. Rather, annotators determined whether each condition was present or absent in the narrative documentation. The NIH notes were manually labeled by a medical student (MN), while the MIMIC notes were labeled by a first-year radiology resident (VB), both under the supervision of a senior radiologist (RMS) with more than 30 years of experience. These binary “yes/no” manual labels were used as the reference standard in the subsequent analysis of performance of AI models.

**Table 1.** Prevalence of the Elixhauser comorbidities in National Institutes of Health (NIH) and Medical Information Mart for Intensive Care (MIMIC) datasets (N=380).

Elixhauser comorbidities	NIH (n=180), n (%)	MIMIC (n=200), n (%)
AIDS/HIV infection	11 (6.1)	1 (0.5)
Alcohol abuse	7 (3.9)	18 (9.0)
Blood loss anemia	1 (0.6)	53 (26.5)
Cardiac arrhythmias	25 (13.9)	63 (31.5)
Chronic pulmonary disease	37 (20.6)	42 (21.0)
Coagulopathy	9 (5.0)	49 (24.5)
Congestive heart failure	4 (2.2)	29 (14.5)
Deficiency anemia	14 (7.8)	82 (41.0)
Depression	31 (17.2)	43 (21.5)
Diabetes	36 (20.0)	31 (15.5)
Drug abuse	1 (0.6)	8 (4.0)
Fluid and electrolyte disorders	35 (19.4)	85 (42.5)
Hypertension	83 (46.1)	109 (54.5)
Hypothyroidism	33 (18.3)	23 (11.5)
Liver disease	46 (25.6)	32 (16.0)
Lymphoma	14 (7.8)	3 (1.5)
Metastatic cancer	33 (18.3)	13 (6.5)
Obesity	19 (10.6)	15 (7.5)
Other neurologic disorders	50 (27.8)	118 (59.0)
Paralysis	2 (1.1)	21 (10.5)
Peptic ulcer disease (excluding bleeding)	8 (4.4)	47 (23.5)
Peripheral vascular disorders	13 (7.2)	10 (5.0)
Psychoses	0 (0)	19 (9.5)
Pulmonary circulation disorders	11 (6.1)	18 (9.0)
Renal failure	12 (6.7)	31 (15.5)
Rheumatoid arthritis/collagen vascular diseases	9 (5.0)	6 (3.0)
Solid tumor without metastasis	70 (38.9)	32 (16.0)
Valvular disease	6 (3.3)	13 (6.5)
Weight loss	23 (12.8)	9 (4.5)

### AI Labeling of Notes

Both datasets were combined, and each note was labeled by each of 46 LLMs, including 4 closed-source models: GPT-4, GPT-4o, Claude 3.5 Sonnet (version 20240620), and Claude 3.5 Sonnet (version 20241022), 13 Llama models (spanning versions 2, 3, 3.1, 3.2, and 3.3), and 29 Qwen models (spanning versions 1, 1.5, 2, and 2.5). Table S1 in Multimedia Appendix 1 provides a full list of the models. Figure S1 in Multimedia Appendix 1 shows an overview of the prompting strategy. The same prompt structure (Figure S1 in Multimedia Appendix 1), asking the model if the patient had a given comorbidity based on the note, was used for every model. An individual prompt was used for each diagnosis and each note; thus, each response corresponded to a unique instance of the tuple (model, note, and diagnosis). If the model refused to answer (due to false-positive content filters in the models) or answered with empty strings, these responses were considered missing and dropped in subsequent analysis. The outputs from the model were also free text, and though the prompt instructed the model to end its answer with yes or no, not all models adhered to the instruction. Therefore, the GPT-o3-mini-high reasoning model was used to analyze the model outputs and parse the answer to a binary yes/no. If the model refused to answer, these were also considered to be missing responses. The prompt used for this step is also reported in Multimedia Appendix 1.

### Performance Measures and Statistical Analysis

The measures used to evaluate performance were recall, precision, and the *F*_1_-score. The metrics and their CIs were evaluated for every model and both datasets. The 95% CIs for precision and recall were computed using the exact Clopper-Pearson method [32]; for the *F*_1_-score, 95% CIs were obtained via a nonparametric bootstrap with 2000 resamples of notes (not individual note-diagnosis pairs, to preserve within-note correlation across diagnoses), recalculating precision, recall, and *F*_1_-score on each resampled dataset and taking the 2.5th and 97.5th percentiles of the resulting *F*_1_-score distribution as the interval bounds.

Additionally, we trained generalized linear models (GLMs) with a binomial family and logit link to predict the recall and precision (separately) using the covariates: model family (Llama, Qwen, GPT, and Claude), log10(model_size) (used for open-weights models only), length of the note (number of words), and the model recency (number of days since the model was released). The full model coefficients are provided in Tables S2 to S5 in Multimedia Appendix 1. The recall model was trained to predict the model response (true/false) on the subset of instances (model, note, and diagnosis), on which the reference standard label was true. Likewise, the precision model was trained to predict the reference standard label on the subset of instances for which the model prediction was true. For the *F*_1_-score, 95% CIs were obtained using the same note-level bootstrap procedure described above, applied to the GLM-derived precision and recall estimates. This was done primarily to analyze the trends of performance for the open-weights models along 2 axes: length of time since the model was released and the model size. This also enabled statistically grounded comparisons of performance across LLM model families while accounting for variability in model size and generation. Additional GLMs (for precision and recall), which also incorporated the dataset and the comorbidities as predictive factors, were trained to explore differences across datasets and diagnoses; for each model family and diagnosis, estimated marginal means of recall and precision were obtained and combined into an *F*_1_-score, with a 95% CI from the same note-level bootstrap procedure described above (Figure S2 and Tables S6 and S7 in Multimedia Appendix 1). Mixed-effects models with random intercepts for the note and the diagnosis were trained to account for the repeated-measures structure (results and Tables S8 and S9 in Multimedia Appendix 1). Because the open-weights models include both text-only and vision-language variants, we additionally tested architecture type (multimodal vs text-only) as a covariate in the recall and precision GLMs to assess whether it confounds the model-family, size, and recency effects. As a check on the fidelity of the quoted evidence supporting each model’s response, we additionally verified whether the quoted text excerpts in each response were actually present in the corresponding note, using an automated string-matching procedure (Table S12 in Multimedia Appendix 1). As a check on the reliability of the GPT-o3-mini-high parsing step, we additionally compared its binary judgments against 3 alternate judge models (GPT-4, GPT-4o, and Claude 3.5 Sonnet) and found high agreement (Table S13 in Multimedia Appendix 1). The analysis was done using R (version 4.3.3; R Foundation for Statistical Computing) and used the emmeans (version 1.10.1; Comprehensive R Archive Network) and dplyr (version 1.1.4; Posit Software, PBC) packages.

### Experimental Setup

All open-weights models, except Llama 3.1 405B, were run on a high-performance computing cluster (Biowulf at the NIH) using the vLLM (version 0.7.3; PyTorch Foundation) package in Python (version 3.12.9; Python Software Foundation). The Llama 3.1 405B and Claude models were run using the Amazon Web Services Bedrock platform. The GPT models were run using the Azure OpenAI Service. The temperature hyperparameter was set to 0 while running the models. The max_tokens parameter, representing the maximum number of tokens to generate per output sequence, was set to 8192 to allow long model outputs.

## Results

### Data

A total of 380 notes, 180 from the NIH and 200 from the MIMIC dataset, were used in this study. The median lengths of a note in the NIH and MIMIC datasets were 1043 (IQR 670-1543) and 1553 (IQR 1106-1948) words, respectively. The prevalence of each Elixhauser comorbidity in each dataset in the reference standard is reported in Table 1.

### Performance of AI Models on the Labeling Task

#### Overall Performance

Overall, 20,036 out of 506,920 (46 models, 29 diagnoses, and 380 notes), 20,204 (3.99%) responses were missing. Of these, 171 (0.85%) missing responses belonged to the 4 closed-source commercial models: GPT-4 accounted for 94 missing responses (87 responses across 3 long notes that exceeded context-length limits, plus 7 unparseable outputs), GPT-4o for 38, Claude 3.5 Sonnet v1 for 6, and Claude 3.5 Sonnet v2 for 33 missing responses.

Table 2 summarizes the precision, recall, and *F*_1_-score (microaveraged over all comorbidities) for all the models. Among the top-performing models were the GPT and Claude models, and some of the larger open-weights models included Llama 3 70B, Llama 3.1 70B, Llama 3.1 405B, Llama 3.3 70B, Qwen 1.5 72B, Qwen 1.5 110B, Qwen 2 72B, and Qwen 2 VL 72B, which all achieve *F*_1_-scores above 70%. The worst performing model was Qwen 2 0.5B, which had an *F*_1_-score of 1.5% (95% CI 0.2-3.2). Macroaveraged *F*_1_-scores are also provided in Table S11 in Multimedia Appendix 1. Rankings by micro- and macroaveraged *F*_1_-scores are highly correlated (Spearman ρ=0.97 across the 46 models).

**Table 2.** Performance of the models in terms of (microaveraged over diagnoses) recall, precision, and *F*_1_-scores.

Model family	Model size (in billions)	Recall (95% CI)	Precision (95% CI)	*F*_1_-score (95% CI)
Claude models				
Claude 3.5 Sonnet v1	—^a^	66.7 (64.4-69.0)	78.8 (76.5-80.9)	72.2 (70.5-73.9)^b^
Claude 3.5 Sonnet v2	—	66.2 (63.9-68.5)	78.5 (76.3-80.6)	71.8 (70.0-73.6)^b^
GPT models				
GPT-4	—	60.9 (58.5-63.3)	83.1 (80.8-85.2)	70.3 (68.4-72.2)^b^
GPT-4o	—	64.4 (62.0-66.7)	78.5 (76.2-80.7)	70.7 (68.8-72.5)^b^
Llama models				
Llama 2	7	91.0 (89.0-92.7)	16.8 (15.8-17.9)	28.4 (26.7-30.1)
Llama 2	13	51.9 (48.7-55.1)	40.4 (37.7-43.2)	45.4 (42.7-48.2)
Llama 2	70	67.0 (64.0-70.0)	45.7 (43.1-48.3)	54.3 (51.8-56.7)
Llama 3	8	83.8 (81.9-85.6)	34.6 (33.2-36.2)	49.0 (47.2-51.1)
Llama 3	70	67.4 (65.0-69.7)	74.6 (72.3-76.8)	70.8 (69.1-72.5)^b^
Llama 3.1	8	75.9 (73.7-77.9)	54.4 (52.4-56.5)	63.4 (61.5-65.1)
Llama 3.1	70	78.5 (76.5-80.5)	65.1 (63.0-67.2)	71.2 (69.7-72.8)^b^
Llama 3.1	405	75.8 (73.7-77.9)	67.6 (65.4-69.7)	71.5 (69.7-73.1)^b^
Llama 3.2	1	89.5 (88.0-91.0)	20.1 (19.2-21.0)	32.8 (31.1-34.4)
Llama 3.2	3	71.0 (68.8-73.2)	42.5 (40.7-44.4)	53.2 (51.5-54.8)
Llama 3.2 VL^c^	11	74.7 (72.6-76.8)	56.7 (54.6-58.8)	64.5 (62.6-66.2)
Llama 3.2 VL	90	73.4 (71.2-75.5)	66.3 (64.1-68.5)	69.7 (67.9-71.4)
Llama 3.3	70	75.6 (73.5-77.7)	69.7 (67.5-71.8)	72.5 (70.9-74.2)^b^
Qwen models				
Qwen 1	1.8	89.1 (87.5-90.5)	17.1 (16.3-17.9)	28.7 (27.3-30.2)
Qwen 1	7	71.6 (69.4-73.8)	25.0 (23.8-26.3)	37.1 (35.3-38.8)
Qwen 1	14	53.0 (48.0-57.9)	65.7 (60.3-70.9)	58.7 (53.8-63.1)
Qwen 1	72	62.3 (59.9-64.7)	76.9 (74.5-79.1)	68.8 (66.9-70.6)
Qwen 1.5	0.5	100.0 (99.8-100.0)	15.1 (14.4-15.8)	26.3 (24.9-27.6)
Qwen 1.5	1.8	98.9 (98.3-99.4)	15.7 (15.0-16.4)	27.1 (25.7-28.4)
Qwen 1.5	4	52.8 (50.4-55.2)	31.8 (30.0-33.5)	39.7 (37.5-42.0)
Qwen 1.5	7	78.0 (76.0-80.0)	45.6 (43.7-47.4)	57.5 (55.5-59.5)
Qwen 1.5	14	75.2 (73.1-77.3)	60.1 (58.0-62.2)	66.8 (65.1-68.5)
Qwen 1.5	32	72.7 (70.5-74.8)	67.3 (65.1-69.5)	69.9 (68.1-71.6)
Qwen 1.5	72	69.0 (66.7-71.2)	75.4 (73.2-77.6)	72.1 (70.4-73.8)^b^
Qwen 1.5	110	70.4 (68.1-72.6)	73.7 (71.5-75.9)	72.0 (70.3-73.7)^b^
Qwen 2	0.5	0.8 (0.4-1.4)	17.8 (9.8-28.5)	1.5 (0.2-3.2)
Qwen 2	1.5	43.0 (40.6-45.4)	66.3 (63.4-69.1)	52.1 (49.6-54.6)
Qwen 2 VL	2	7.5 (6.3-8.9)	90.6 (84.4-94.9)	13.9 (11.5-16.5)
Qwen 2	7	77.0 (74.9-79.0)	57.4 (55.3-59.5)	65.8 (63.9-67.7)
Qwen 2 VL	7	55.3 (52.9-57.7)	79.5 (77.0-81.8)	65.2 (63.2-67.3)
Qwen 2	72	68.5 (66.2-70.7)	76.7 (74.5-78.9)	72.4 (70.6-74.0)^b^
Qwen 2 VL	72	62.3 (59.9-64.6)	81.2 (78.9-83.3)	70.5 (68.7-72.3)^b^
Qwen 2.5	0.5	74.3 (72.1-76.4)	21.6 (20.6-22.7)	33.5 (31.9-35.2)
Qwen 2.5	1.5	42.5 (40.1-44.9)	77.1 (74.3-79.8)	54.8 (52.3-57.3)
Qwen 2.5	3	50.7 (48.3-53.2)	64.4 (61.8-67.0)	56.8 (54.5-59.0)
Qwen 2.5 VL	3	75.0 (72.9-77.1)	29.0 (27.7-30.4)	41.9 (39.8-43.9)
Qwen 2.5	7	60.9 (58.5-63.3)	73.0 (70.6-75.3)	66.4 (64.5-68.3)
Qwen 2.5 VL	7	63.0 (60.6-65.3)	65.0 (62.6-67.3)	64.0 (62.0-65.9)
Qwen 2.5	14	56.8 (54.4-59.2)	80.9 (78.5-83.1)	66.8 (64.8-68.7)
Qwen 2.5	32	60.4 (58.0-62.7)	80.9 (78.6-83.0)	69.1 (67.2-71.0)
Qwen 2.5	72	58.4 (56.0-60.8)	80.9 (78.6-83.1)	67.9 (65.8-69.8)
Qwen 2.5 VL	72	60.9 (58.5-63.3)	80.2 (77.9-82.4)	69.3 (67.4-71.0)

a Not available.

b Denotes *F*_1_-scores above 70%.

c VL: vision language.

#### Sensitivity Analysis for Missing Responses

Missing/unparseable responses were not evenly distributed across models: 4 open-weights models accounted for a disproportionate share of them—Qwen 1 14B (65.0% missing), Llama 2 13B (31.0%), Llama 2 70B (30.8%), and Llama 2 7B (30.7%)—while the remaining 42 models each had a missing rate below 2.8%. If missing responses are instead treated as mistakes rather than dropped (a false negative when the true label was positive, a false positive when it was negative), the performance of these 4 models drops substantially: *F*_1_-score falls from 58.7% to 5.4% for Qwen 1 14B, from 45.4% to 17.9% for Llama 2 13B, from 54.3% to 22.5% for Llama 2 70B, and from 28.4% to 18.4% for Llama 2 7B. For the remaining 42 models, where the missing rate was at most 2.8%, the median observed drop in *F*_1_-score was 0.6 (IQR 0.2‐0.9, max: 5.8) percentage points. This confirms that dropping missing responses has a negligible effect on the reported results overall, except for these 4 models with unusually high refusal/nonparse rates, whose performance should be interpreted with this caveat in mind.

### Performance Modeling and Trends

#### Comparing Model Families

Figure 1 shows the average performance across model families for both closed-source and open-weights models. Among closed-source model families, Claude showed higher recall than GPT (estimated marginal means: 65.9 vs 63.3; *P*=.04) but similar mean precision (79.4% vs 80.0; *P*=.60), resulting in higher *F*_1_-score (72.0 vs 70.6). These differences are unlikely to be clinically relevant. For the open-weights models, Llama had higher average recall (76.2 vs 62.6; *P*<.001) but lower average precision (29.5 vs 42.3; *P*<.001), resulting in a lower overall *F*_1_-score (42.5 vs 50.5). The figure represents the estimated marginal means, which are adjusted for covariates such as model size and recency.

**Figure 1.**
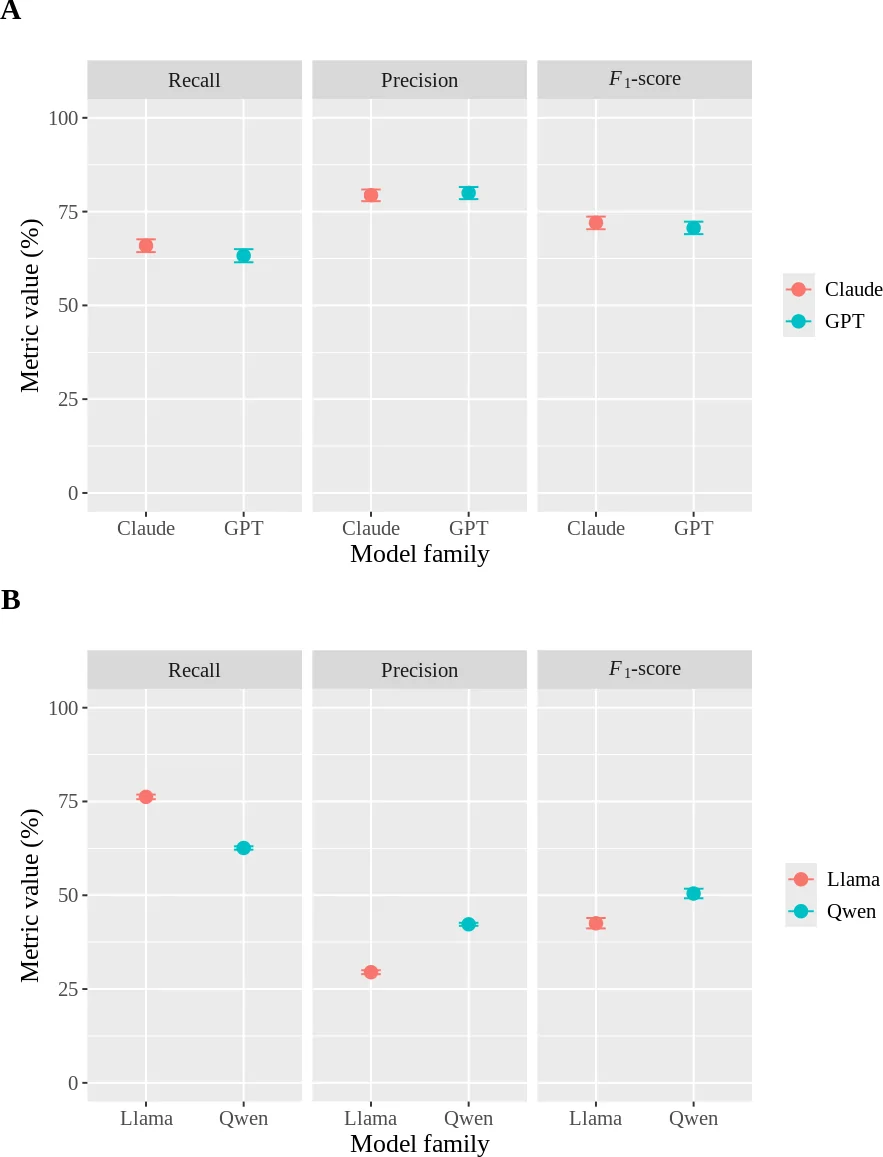
Comparing performance across model families for (A) closed-source and (B) open-weights models.

#### Effect of Model Size

Figure 2 shows the effect of model size on the performance of open-weights models. As is clear from the figure, recall shows a small but statistically significant increase (estimated coefficient of log10(model_size): 0.055; *P*<.001) with increasing model size, but the precision (and consequently, *F*_1_-score) shows a substantially larger upward trend with increasing model size (coefficient: 1.275; *P*<.001).

**Figure 2.**
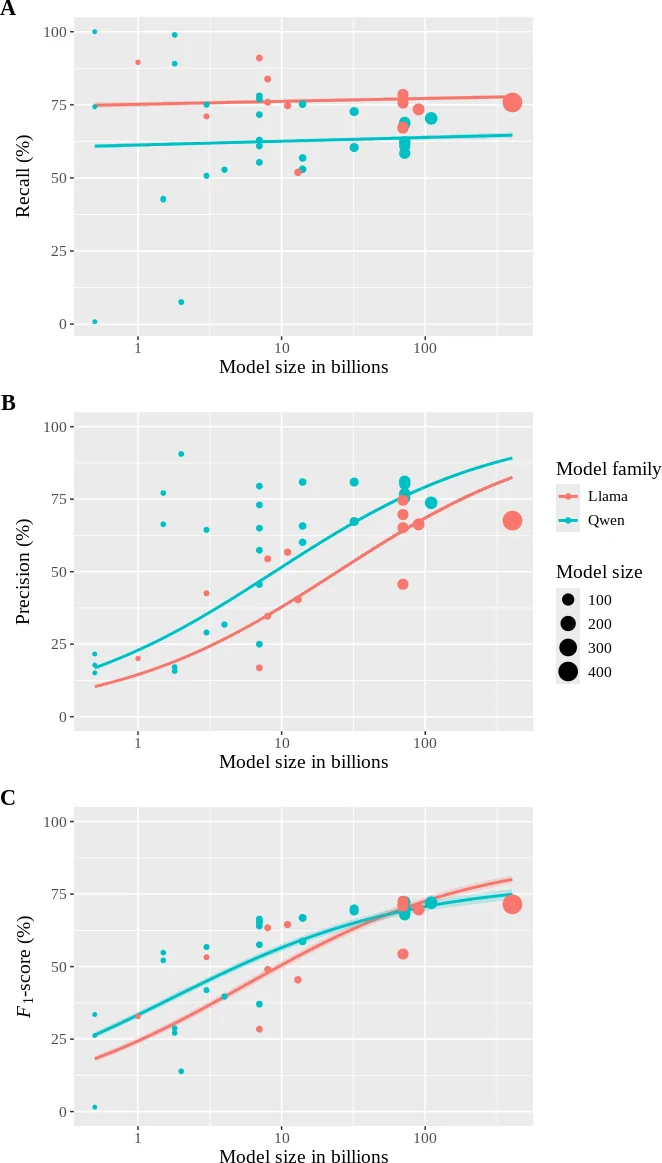
Performance of the open-weights models with model size, in terms of (A) recall, (B) precision, and (C) *F*_1_-score. The regression line is obtained from generalized linear models for recall and precision, fit on response data using predictors including model family, duration since model release, note length in words, and the size of the model.

#### Effect of Model Recency

Figure 3 shows the trend of model performance over time. As is clear from the figure, the closed-source models have little variation in *F*_1_-scores with respect to the release recency of the model. On the other hand, for the open-weights models, strong trends are observed: the recall of the models has tended to decrease, while the precision of the models has gone up, resulting in an improvement of *F*_1_-scores for the more recent models.

**Figure 3.**
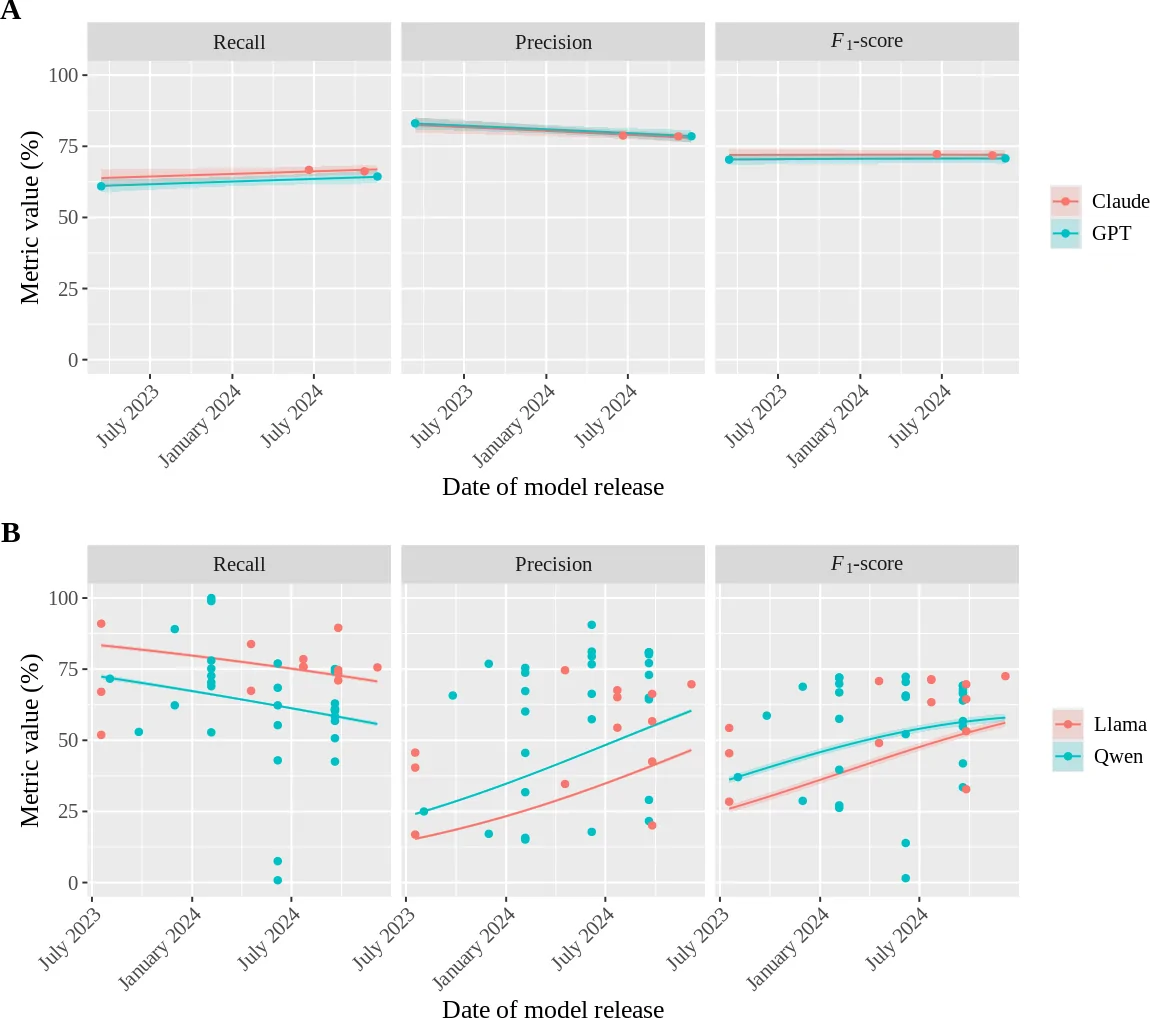
Performance of the (A) closed-source and (B) open-weights model vs date of model response. Note that the regression line is obtained from generalized linear models for recall and precision, fit on response data using predictors including model family, duration since model release, note length and words, and the size of the model (for open-weights models only). For the closed-source models, the trend lines are fit across only 2 models per family (GPT-4/GPT-4o and Claude 3.5 Sonnet v1/v2) and should be interpreted as illustrative rather than a statistically robust trend.

#### Effect of Note Length

Figure 4 shows the effect of note length on the performance of the models. The closed-source models show consistent performance irrespective of the length of the note. The open-weights models, on the other hand, show improved performance with increasing note length. This finding contradicts previous reports in the literature [7] and underscores the need for further investigation. In this analysis, all variables except note_length were held constant in the GLMs in order to control for model factors such as size and recency.

**Figure 4.**
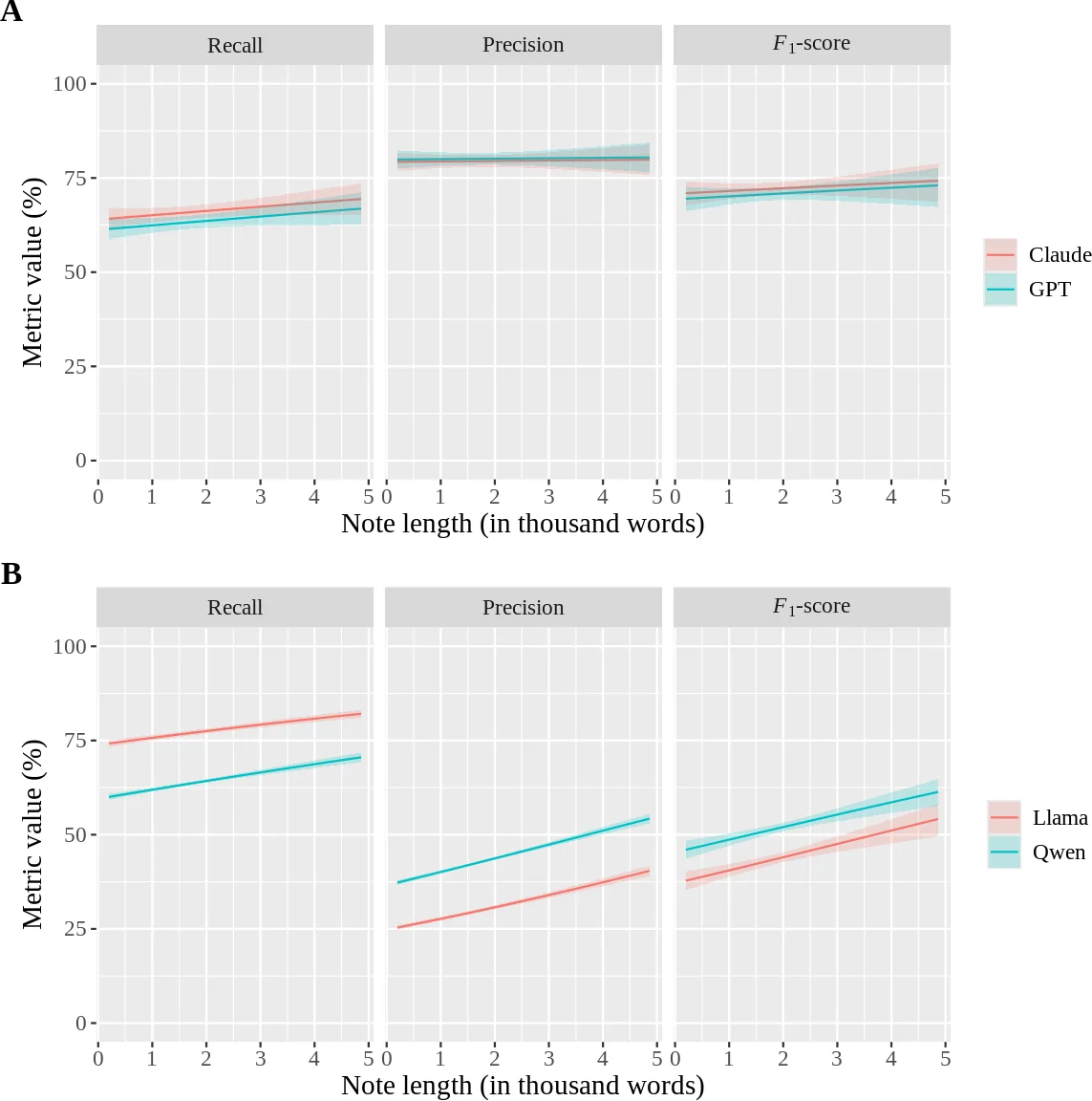
Performance of the (A) closed-source models and (B) open-weights models vs the length of the notes. For the closed-source models, the trend lines are fit across only 2 models per family (GPT-4/GPT-4o and Claude 3.5 Sonnet v1/v2) and should be interpreted as illustrative rather than a statistically robust trend.

#### Effect of Model Architecture

Because the open-weights model set pools text-only and vision-language (multimodal) variants of the same architectures, we tested architecture type as an additional covariate in the open-weights recall and precision GLMs. Multimodal architecture was a significant negative predictor of both recall and precision (both *P*<.001; Table S10 in Multimedia Appendix 1), indicating that vision-language variants underperform their text-only counterparts of the same family, size, and release date on this text-only clinical extraction task. Including this covariate did not change the sign or significance of the model-family, size, or recency effects reported above, indicating those associations are not confounded by the mix of architectures among the open-weights models.

#### Variation of Performance Across Different Comorbidities

Figure S2 in Multimedia Appendix 1 shows the average performance of the model families across different comorbidities. Among the open-weights models, performance was worst for drug abuse (estimated mean *F*_1_-scores of 11.6%, 95% CI 4.8‐18.6 for Llama and 18.2%, 95% CI 7.9‐27.8 for Qwen); the closed-source models performed markedly better on this comorbidity (77.0%, 95% CI 51.8‐89.8 for Claude and 76.2%, 95% CI 51.1‐89.4 for GPT). The best performance across all 4 model families was obtained on hypertension, with estimated mean *F*_1_-scores of 76.7% (95% CI 72.7‐80.3) for Llama and 80.0% (95% CI 77.2‐82.4) for Qwen and 93.3% (95% CI 90.8‐95.5) for Claude and 92.9% (95% CI 90.3‐95.1) for GPT. For AIDS/HIV infection, Claude and GPT achieved an *F*_1_-score of 100% reflecting perfect agreement with the reference standard on all 12 positive cases for this comorbidity in our dataset.

## Discussion

### Principal Findings

In this study, we benchmarked the performance of 46 LLMs, including 4 closed-source commercial models (GPT-4, GPT-4o, Claude 3.5 Sonnet [version 20240620] and Claude 3.5 Sonnet [version 20241022]), and 42 open-weights LLMs from 2 popular model families, Llama and Qwen, spanning various model sizes and generations. Using GLMs, we also analyzed how the performance varied based on various factors such as the model size, family, and generation and the length of the notes. We found that in the labeling task, larger open-weights models such as the Llama 3.3 70B and the Qwen 2 72B performed at par or better than the commercial models in terms of *F*_1_-scores. Controlling for model factors such as size and recency, the Qwen family had higher precision and *F*_1_-scores than the Llama family of models. Model size primarily improved precision (recall showed much smaller improvement), leading to an overall increase in *F*_1_-scores. Recent open-weights models have lower recall but significantly higher precision than older models, resulting in an improvement in *F*_1_-scores. Perhaps surprisingly, longer notes seemed to improve the performance of open-weights models (the closed-source models had a relatively stable performance) in terms of both recall and precision.

### Comparison to Prior Work

The performance of LLMs on similar tasks has been explored in prior literature. For example, several papers have studied the task of note summarization and found that LLMs can perform on par with clinicians, with low hallucination and omission rates [33,34]. Other papers have tackled the task of labeling clinical notes; for example, Burford et al [20] evaluated the performance of GPT-4 in identifying helmet status of patients injured in micromobility-related accidents from clinical notes, evaluating the extraction of presence of a single condition. Wu et al [7] extracted a variety of conditions from clinical notes but did not report performance for individual conditions, likely due to the rarity of the target diseases. McInerney et al [6] extracted 10 common conditions but relied on a single, now-outdated LLM, achieving an average *F*_1_-score of approximately 0.42. Other papers have focused on labeling other forms of clinical text such as radiology or pathology reports [4,35]; however, clinical notes considered in this study are usually longer and pose greater challenges than radiology and pathology reports. Most existing papers focus on single LLMs or single conditions, unlike this current study that uses a multi-institutional set of notes, a diverse set of comorbidities to be labeled, and evaluates 46 models, including 2 families of closed-source commercial LLMs (GPT and Claude) and 2 families of open-weights LLMs (Llama and Qwen). Additionally, we modeled the performance of these LLMs using factors such as model family, size, and recency to provide insight into how these factors affect performance.

While it is somewhat dogmatic to assume that newer and larger model sizes will necessarily be better at every task, it may not always be the case. For example, Zhou et al [36] found that larger and more instructable models may become less reliable. Our results show the general trend of improving performance with increasing model size holds for the note-labeling task. However, outliers exist, and, in some cases, the general trend may not be true; for example, the recall of Llama 3.1 405B is less than that of the Llama 3 8B model (75.8, 95% CI 73.7-77.9 vs 83.8, 95% CI 81.9-85.6). When considering model recency, we found that the recall shows a decreasing trend—recent models have lower recall than older models (though this was accompanied by an improvement in precision and an improvement in the overall *F*_1_-score). Notably, within the Qwen family, Qwen 2.5 72B showed lower recall than Qwen 2 72B (58.4% vs 68.5%), offset by higher precision (80.9% vs 76.7%), reducing the overall *F*_1_-score (67.9% vs 72.4%) in the newer generation model.

One of the surprising findings in the study was that the open-weights LLMs tended to perform better (both precision and recall) on longer notes when controlling for model factors such as family, size, and recency. The effect did not go away when controlling for the dataset (Tables S6 and S7 in Multimedia Appendix 1), and in fact, the dataset was not a significant factor when modeling precision for the open-weights models. This may perhaps be due to more redundancy or more explicit presentation of information in longer notes.

In this study, we chose to combine the datasets from 2 different institutions (n=180 and n=200) to create a single large cohort (n=380) on which all the models were tested. This increased the diversity of the notes, both note type and institution (history and physical notes from the NIH and discharge notes from the MIMIC datasets), as well as the size of the cohort, giving us more robust estimates of real-world performance and trends. We also did not use the dataset or note type as factors in the GLMs. This simplicity allowed us to focus on the model factors such as family, size, and recency and analyze how those influence performance. Additionally, an alternative formulation of the GLMs with dataset (consequently the note type, as well) for the open-weights models shows that the coefficients (and trends) of the fitted models are very similar, particularly for the model-related factors (note length was significantly associated with the dataset; Tables S6 and S7 in Multimedia Appendix 1). This also highlights the robustness of the GLM-based analysis method used here, a conclusion further supported by a mixed-effects model sensitivity analysis accounting for repeated measures within notes and diagnoses (Tables S8 and S9 in Multimedia Appendix 1).

### Limitations

Our study has several limitations. First, the reference standard was established by a medical student and a first-year radiology resident under the supervision of a senior radiologist, rather than by internal medicine physicians or certified medical coders. Although the annotation task involved simplified binary identification of clinical conditions in narrative notes rather than formal Elixhauser coding or billing-code assignment, some conditions may have required greater specialty-specific or coding expertise. In addition, the annotations were completed in sequential phases without a double-labeled subset, precluding formal assessment of interrater reliability. The senior radiologist provided guidance and spot-checked the annotations, but this quality-control process does not substitute for specialist adjudication or quantitative measurement of agreement. Consequently, some degree of reference-standard misclassification cannot be excluded. Second, the GLMs used to model trends in model family, size, and recency (Figures 2 and 3) exclude comorbidity as a predictor, in favor of the interpretability of those specific coefficients. A separate analysis incorporating comorbidity (Figure S2 in Multimedia Appendix 1) confirmed substantial variation in performance across conditions, underscoring the importance of evaluating LLMs on the specific clinical use case before deployment. Third, since the MIMIC-IV-Note dataset is publicly available, it may have been included, in part or whole, in the training corpora of at least some of the LLMs we evaluated, and this may have affected the reported performance. However, including the dataset as an additional covariate in the GLMs for precision and recall did not alter the coefficients of model family, model size, and model recency (note length showed more modest shifts), suggesting that the principal associations identified in our analyses were robust to adjustment for systematic differences between the public MIMIC-IV-Note dataset and the nonpublic institutional dataset. Fourth, we used a fixed prompt for all our models and did not evaluate the variability of results to variation in the prompts. Additionally, the prompt did not explicitly instruct models to distinguish between comorbidities active at the time of admission and those noted only as part of the patient’s past history, and models may not have reliably made this distinction on their own. Fifth, our automated parsing of free-text responses into binary judgments, while shown to have high agreement across independent judge models (Table S13 in Multimedia Appendix 1), was not manually verified at scale and cannot rule out an error shared by all LLM judges—for example, a note phrasing that misleads every judge model in the same way. Finally, we dropped missing/unparseable responses (3.99% overall) from our analysis. The median model had fewer than 0.4% missing responses, but 4 models—Qwen 1 14B (65.0%) and the 3 Llama 2 variants (30.7%‐31.0%)—had substantially higher rates. A sensitivity analysis recomputing performance with missing responses treated as incorrect shows that this had negligible effect for nearly all models, but shifted *F*_1_-score by 10 to 53 percentage points for these 4 high-missingness models. We therefore expect our reported performance to be reliable for most models, with the results for these 4 specifically interpreted with this caveat in mind.

### Conclusions

We performed a comprehensive evaluation of 2 families of closed-source commercial LLMs (GPT and Claude) and 2 families of popular open-weights LLMs (Llama and Qwen) on the task of labeling clinical notes. We also analyzed the trends of performance with respect to various factors such as model family, model size, and model recency. Our results present a comprehensive picture of the LLM performance on the clinically relevant task of labeling clinical notes.

## Supplementary material

10.2196/81708 Multimedia Appendix 1 Supplementary methodological detail, additional modeling approaches, and per-comorbidity performance results, including Tables S1–S13 and Figures S1–S2.

## Abbreviations

ECI: Elixhauser Comorbidity Index
GLM: generalized linear model
ICD: International Classification of Diseases
LLM: large language model
MIMIC: Medical Information Mart for Intensive Care
NIH: National Institutes of Health
NLP: natural language processing
VL: vision language
