# Supplementary material for: Labeling Clinical Notes With Large Language Models: Retrospective Benchmarking Study
Source: J Med Internet Res. 2026 Nov 10;28:e81708. doi: 10.2196/81708 (PMC13756136; doi:10.2196/81708)
Supplement: Supplementary file 1 [file jmir-v28-e81708-s001.pdf]

Multimedia Appendix 1

Prompt Structure

There were two prompt structures used in the study. First, the following prompt (some formatting characters such as newline have been removed) was used to get the initial free-text responses of the models.

"You are a helpful physician assistant. I will give you an anonymized clinical note and ask you if the patient has a disease or condition. Please read the note carefully, think step by step, and answer yes or no. If the condition is not mentioned at all (either directly or indirectly), you can answer no. If yes, and you think the patient does have the disease or condition, please extensively quote all those text excerpts which support your conclusion. Here is the note:"

+

Clinical Note

+

"Does the patient have Congestive heart failure? Think step by step and provide evidence for your response. Finally, summarize your reasoning and end your answer with YES or NO."

In the next step, the GPT-o3-mini model was used to parse the free text responses of the model to a binary yes/no answer. The prompt used for this task was:

"I will give you the following text which describes if there is evidence of a patient having a certain disease."

+

Free text model response

+

"Now please summarize the conclusion of the text and pick one of two choices: (A) YES, or (B) NO. Please reply (A) or (B) only"

Models used in this study

Table S1:Models used in this study. The Model Id for the open-weights models are obtained from Huggingface; for GPT and Claude, the ids from Azure OpenAI and from AWS Bedrock are used.

Model name	Model Id	Multimodal†	Open weights	Date of release*
Claude 3.5 Sonnet v1	claude-3-5-sonnet-20240620	yes	no	6/20/2024
Claude 3.5 Sonnet v2	claude-3-5-sonnet-20241022	yes	no	10/22/2024
GPT-4	gpt-4	yes	no	3/14/2023
GPT-4o	gpt-4o-2024-11-20	yes	no	11/20/2024
Llama 2 7B	meta-llama/Llama-2-7b-chat-hf	no	yes	7/18/2023

Llama 2 13B	meta-llama/Llama-2-13b-chat-hf	no	yes	7/18/2023
Llama 2 70B	meta-llama/Llama-2-70b-chat-hf	no	yes	7/18/2023
Llama 3 8B	meta-llama/Meta-Llama-3-8B-Instruct	no	yes	4/18/2024
Llama 3 70B	meta-llama/Meta-Llama-3-70B-Instruct	no	yes	4/18/2024
Llama 3.1 8B	meta-llama/Llama-3.1-8B-Instruct	no	yes	7/23/2024
Llama 3.1 70B	meta-llama/Llama-3.1-70B-Instruct	no	yes	7/23/2024
Llama 3.1 405B	meta-llama/Llama-3.1-405B-Instruct	no	yes	7/23/2024
Llama 3.2 1B	meta-llama/Llama-3.2-1B-Instruct	no	yes	9/25/2024
Llama 3.2 3B	meta-llama/Llama-3.2-3B-Instruct	no	yes	9/25/2024
Llama 3.2 VL 11B	meta-llama/Llama-3.2-11B-Vision-Instruct	yes	yes	9/25/2024
Llama 3.2 VL 90B	meta-llama/Llama-3.2-90B-Vision-Instruct	yes	yes	9/25/2024
Llama 3.3 70B	meta-llama/Llama-3.3-70B-Instruct	no	yes	12/6/2024
Qwen 1 1.8B	Qwen/Qwen-1_8B-Chat	no	yes	11/30/2023
Qwen 1 7B	Qwen/Qwen-7B-Chat	no	yes	8/3/2023

Qwen 1 14B	Qwen/Qwen-14B-Chat	no	yes	9/25/2023
Qwen 1 72B	Qwen/Qwen-72B-Chat	no	yes	11/30/2023
Qwen 1.5 0.5B	Qwen/Qwen1.5-0.5B-Chat	no	yes	2/5/2024
Qwen 1.5 1.8B	Qwen/Qwen1.5-1.8B-Chat	no	yes	2/5/2024
Qwen 1.5 4B	Qwen/Qwen1.5-4B-Chat	no	yes	2/5/2024
Qwen 1.5 7B	Qwen/Qwen1.5-7B-Chat	no	yes	2/5/2024
Qwen 1.5 14B	Qwen/Qwen1.5-14B-Chat	no	yes	2/5/2024
Qwen 1.5 32B	Qwen/Qwen1.5-32B-Chat	no	yes	2/5/2024
Qwen 1.5 72B	Qwen/Qwen1.5-72B-Chat	no	yes	2/5/2024
Qwen 1.5 110B	Qwen/Qwen1.5-110B-Chat	no	yes	2/5/2024
Qwen 2 0.5B	Qwen/Qwen2-0.5B-Instruct	no	yes	6/6/2024
Qwen 2 1.5B	Qwen/Qwen2-1.5B-Instruct	no	yes	6/6/2024
Qwen 2 VL 2B	Qwen/Qwen2-VL-2B-Instruct	yes	yes	6/6/2024
Qwen 2 7B	Qwen/Qwen2-7B-Instruct	no	yes	6/6/2024
Qwen 2 VL 7B	Qwen/Qwen2-VL-7B-Instruct	yes	yes	6/6/2024
Qwen 2 72B	Qwen/Qwen2-72B-Instruct	no	yes	6/6/2024
Qwen 2 VL 72B	Qwen/Qwen2-VL-72B-Instruct	yes	yes	6/6/2024

Qwen 2.5 0.5B	Qwen/Qwen2.5-0.5B-Instruct	no	yes	9/19/2024
Qwen 2.5 1.5B	Qwen/Qwen2.5-1.5B-Instruct	no	yes	9/19/2024
Qwen 2.5 3B	Qwen/Qwen2.5-3B-Instruct	no	yes	9/19/2024
Qwen 2.5 VL 3B	Qwen/Qwen2.5-VL-3B-Instruct	yes	yes	9/19/2024
Qwen 2.5 7B	Qwen/Qwen2.5-7B-Instruct	no	yes	9/19/2024
Qwen 2.5 VL 7B	Qwen/Qwen2.5-VL-7B-Instruct	yes	yes	9/19/2024
Qwen 2.5 14B	Qwen/Qwen2.5-14B-Instruct	no	yes	9/19/2024
Qwen 2.5 32B	Qwen/Qwen2.5-32B-Instruct	no	yes	9/19/2024
Qwen 2.5 72B	Qwen/Qwen2.5-72B-Instruct	no	yes	9/19/2024
Qwen 2.5 VL 72B	Qwen/Qwen2.5-VL-72B-Instruct	yes	yes	9/19/2024

* The dates are in the mm/dd/yyyy format.

† Multimodal indicates that the model is a vision-language model, i.e., it can handle both images and text.

Recall and Precision Models

We fitted separate models for precision and recall for closed and open-weights models in this study. The coefficients of the fitted GLM models are reported below:

Closed-Source Models

Recall model

Table S2: Recall GLM for closed-source LLMs

term	estimate	std.error	p.value
(Intercept)	0.6610573	0.0704536	<.001
model_familyGPT	-0.116708	0.0573076	0.042
note_length	5.04155e-05	2.924030e-05	0.085
duration_since_release	-0.000220	0.0001145	0.055

Precision model

Table S3: Precision GLM for close-source LLMs

term	estimate	std.error	p.value
(Intercept)	1.156866	0.090841	<.001
model_familyGPT	0.038026	0.073390	0.604
note_length	0.000007	0.000038	0.844
duration_since_release	0.000464	0.000160	0.004

Open-Weights Models

Recall model

Table S4: Recall GLM for open-weights LLMs

term	estimate	std.error	p.value

(Intercept)	0.392300	0.035924	<.001
model_familyQwen	-0.650114	0.020183	<.001
log10_model_size	0.055251	0.011526	<.001
note_length	0.000100	0.000010	<.001
duration_since_release	0.001444	0.000068	<.001

Precision model

Table S5: Precision GLM for open-weights LLMs

term	estimate	std.error	p.value
(Intercept)	-0.711339	0.027535	<.001
model_familyQwen	0.559542	0.015592	<.001
log10_model_size	1.274732	0.010312	<.001
note_length	0.000148	0.000008	<.001
duration_since_release	-0.003096	0.000049	<.001

GLMs for open-weights models when using the dataset as a predictive factor

In our study, the two datasets were combined into one cohort and the GLMs were trained without considering the dataset as a factor influencing performance. Here we present alternative GLMs fitted considering the dataset as a predictive factor. Note the similarity of the coefficients with and without considering the dataset as a factor, especially for factors other than note_length, which is correlated with the dataset.

Recall model

Table S6: Recall GLM for open-weights LLMs with dataset as an additional covariate

term	estimate	std.error	p.value
(Intercept)	0.138129	0.037561	<.001
model_familyQwen	-0.657249	0.020276	<.001
log10_model_size	0.055377	0.011585	<.001
note_length	0.000151	0.000010	<.001
duration_since_release	0.001455	0.000068	<.001
datasetNIH	0.458207	0.017896	<.001

Precision model

Table S7: Precision GLM for open-weights LLMs with dataset as an additional covariate

term	estimate	std.error	p.value
(Intercept)	-0.705666	0.028567	<.001
model_familyQwen	0.559699	0.015593	<.001
log10_model_size	1.275126	0.010326	<.001
note_length	0.000147	0.000008	<.001
duration_since_release	-0.003095	0.000049	<.001
datasetNIH	-0.010584	0.014198	0.46

Mixed-effects models with note and diagnosis as random effects

Table S8: Random-effect variances (SD) from generalized linear mixed models with random intercepts for note and diagnosis, added to each of the four generalized linear models reported in the main text.

Outcome model	Note variance (SD)	Diagnosis variance (SD)
Closed-source — Recall	1.10 (1.05)	2.26 (1.50)
Closed-source — Precision	1.92 (1.38)	1.20 (1.09)
Open-weights — Recall	0.47 (0.69)	0.62 (0.78)
Open-weights — Precision	0.73 (0.85)	0.66 (0.81)

Table S9: Fixed-effect estimates from the generalized linear models (GLM) versus the generalized linear mixed models (GLMM) with random intercepts for note and diagnosis. ***Note: Continuous covariates are standardized (mean 0, SD 1) in both models for direct comparability.***

Model	Term	GLM est. (SE), *P*	GLMM est. (SE), *P*
Closed — Recall	Model family (GPT vs Claude)	−0.117 (0.057), .042	−0.169 (0.069), .015
	Note length	0.045 (0.026), .085	0.147 (0.068), .031
	Model recency	−0.054 (0.028), .055	−0.080 (0.034), .020

Model	Term	GLM est. (SE), *P*	GLMM est. (SE), *P*
Closed — Precision	Model family (GPT vs Claude)	0.038 (0.073), .604	0.065 (0.084), .440
	Note length	0.007 (0.034), .844	0.065 (0.093), .486
	Model recency	0.111 (0.038), .004	0.111 (0.043), .011
Open — Recall	Model family (Qwen vs Llama)	−0.650 (0.020), <.001	−0.768 (0.022), <.001
	log10(model size)	0.041 (0.009), <.001	0.048 (0.009), <.001
	Note length	0.089 (0.008), <.001	0.134 (0.039), <.001
	Model recency	0.189 (0.009), <.001	0.225 (0.010), <.001
Open — Precision	Model family (Qwen vs Llama)	0.560 (0.016), <.001	0.556 (0.017), <.001
	log10(model size)	0.943 (0.008), <.001	0.898 (0.009), <.001
	Note length	0.127 (0.007), <.001	0.237 (0.046), <.001
	Model recency	−0.445 (0.007), <.001	−0.442 (0.008), <.001

GLMs with architecture-type (multimodal vs text-only) on the open-weight models

The open-weights model set pools text-only models with vision-language (multimodal) variants of the same architectures (Llama 3.2 VL, Qwen2 VL, Qwen2.5 VL; the four closed-source

models are all multimodal, so this does not apply to the closed-source GLMs). To check whether this introduces unaccounted-for variance, we refit the open-weights recall and precision GLMs reported in the main text with an added binary covariate for architecture type (Supplementary Table 10). Multimodal architecture was a significant negative predictor of both recall and precision (both $p < 0.001$ by likelihood-ratio test), indicating that vision-language variants underperform their text-only counterparts of the same family, size, and release date on this text-only clinical extraction task. Variance inflation factors for the multimodal covariate were low (1.13 for recall, 1.26 for precision), indicating it is not collinear with model family, size, note length, or release duration. Including it shifts the other coefficients only modestly and does not change their sign or significance.

Table S10: Model coefficients for the GLMs, without and with the "multimodal" covariate. Multimodal open-weight models show a significant reduction in performance ($P < .001$) compared to their text-only counterparts, when controlled for model size and recency.

Outcome	Term	Published GLM	+ Multimodal covariate
Recall	Intercept	0.393	0.526
Recall	Model family (Qwen vs. Llama)	-0.650	-0.620
Recall	log10(model size)	0.055	0.074
Recall	Multimodal (yes vs. no)	--	-0.284 ($p < 0.001$)
Recall	Note length	9.99e-05	9.92e-05

Outcome	Term	Published GLM	+ Multimodal covariate
Recall	Duration since release	1.44e-03	1.14e-03
Precision	Intercept	-0.711	-0.627
Precision	Model family (Qwen vs. Llama)	0.560	0.583
Precision	log10(model size)	1.275	1.296
Precision	Multimodal (yes vs. no)	--	-0.197 ($p < 0.001$)
Precision	Note length	1.48e-04	1.46e-04
Precision	Duration since release	-3.10e-03	-3.30e-03

Macro-averaged (over comorbidities) performance of models

Table S11: Macro-averaged precision, recall, and F_1 score (averaged equally across the 29 comorbidities, unlike the micro-averaged Table 2) for each of the 46 models. "Positives / Valid responses" gives the total number of responses the model judged positive (true positives + false positives) and the total number of parseable (non-missing) responses across all 29 diagnoses for that model.

Model	Positives / Valid responses	Precision (%)	Recall (%)	F_1 (%)
Claude 3.5 Sonnet v2	1400 / 10987	76.4	67.7	69.7
Llama 3.3 70B	1798 / 10973	67.3	76.4	69.6

Model	Positives / Valid responses	Precision (%)	Recall (%)	F_1 (%)
Llama 3.1 405B	1864 / 11000	66.1	78.2	69.5
Qwen 2 72B	1482 / 10989	73.9	70.1	69.5
Qwen 1.5 72B	1523 / 11017	74.2	70.3	69.4
Qwen 1.5 110B	1584 / 10968	72.1	71.9	69.3
Claude 3.5 Sonnet v1	1409 / 11014	76.7	66.9	69.1
GPT-4o	1363 / 10982	76.5	66.9	68.8
Llama 3.1 70B	2000 / 10981	62.6	78.3	67.9
GPT-4	1206 / 10926	80.3	62.8	67.8
Qwen 2 VL 72B	1276 / 11014	76.8	65.6	67.7
Qwen 2.5 32B	1240 / 11003	79.7	64.6	67.5
Qwen 2.5 VL 72B	1257 / 10948	78.4	64.9	67.4
Qwen 1.5 32B	1795 / 10967	64.4	75.2	67.1
Qwen 1 72B	1324 / 10748	75.6	65.1	66.9
Llama 3 70B	1470 / 10861	71.1	68.2	66.9
Llama 3.2 VL 90B	1843 / 11002	64.2	74.3	66.7
Qwen 2.5 72B	1200 / 11011	79.2	63.1	66.5

Model	Positives / Valid responses	Precision (%)	Recall (%)	F_1 (%)
Qwen 2.5 7B	1388 / 11009	70.8	63.6	64.7
Qwen 1.5 14B	2074 / 10983	60.7	73.6	63.6
Qwen 2.5 14B	1168 / 11007	79.2	59.4	63.6
Llama 3.2 VL 11B	2190 / 10998	56.6	76.0	62.3
Qwen 2 VL 7B	1151 / 10978	76.1	57.3	62.2
Qwen 2 7B	2190 / 10831	56.7	74.5	61.7
Qwen 2.5 VL 7B	1611 / 11013	61.8	66.6	61.5
Llama 3.1 8B	2306 / 10958	53.7	76.9	60.8
Qwen 2.5 3B	1304 / 10947	63.3	52.7	54.1
Qwen 1.5 7B	2829 / 10975	45.1	77.4	54.0
Qwen 2.5 1.5B	918 / 11015	76.7	43.7	51.2
Qwen 2 1.5B	1075 / 10981	67.8	49.1	51.1
Llama 3.2 3B	2774 / 10931	41.1	71.2	49.5
Llama 3 8B	3934 / 10786	32.8	83.6	43.9
Llama 2 70B	1421 / 7629	46.6	39.8	40.7
Qwen 2.5 VL 3B	4245 / 10895	27.7	75.4	37.4

Model	Positives / Valid responses	Precision (%)	Recall (%)	F_1 (%)
Qwen 1.5 4B	2767 / 11017	30.6	53.0	35.9
Qwen 1 7B	4710 / 10887	22.9	68.5	32.3
Llama 2 13B	1250 / 7600	36.2	30.9	31.0
Qwen 2.5 0.5B	5703 / 10979	21.1	75.3	30.5
Llama 3.2 1B	7370 / 10891	19.2	88.1	29.7
Qwen 1 1.8B	8476 / 10813	16.7	88.1	26.3
Qwen 1.5 1.8B	10448 / 10974	15.6	99.0	25.3
Qwen 1.5 0.5B	11018 / 11018	15.1	100.0	24.7
Llama 2 7B	5223 / 7638	15.7	52.6	22.6
Qwen 1 14B	327 / 3859	56.0	13.8	20.9
Qwen 2 VL 2B	138 / 11007	75.0	9.6	15.6
Qwen 2 0.5B	73 / 10706	15.9	0.8	1.5

Estimating hallucinations: Verification of quoted evidence in model responses

The prompt used in this study asked each model to extensively quote text excerpts from the clinical note supporting its conclusion, but only the final binary judgment was scored in the main analysis. To assess whether models occasionally reached a correct answer despite citing unsupported or fabricated evidence, we performed an automated check: for each response, all quoted spans (text enclosed in quotation marks) were extracted and compared against the corresponding note, after normalizing whitespace and markdown formatting, stripping trailing punctuation introduced by truncation, and excluding quotes that merely restated the diagnosis name or the question itself rather than citing note content. A quote was considered "verified" if it (or, for quotes containing an ellipsis, each of its segments) appeared verbatim in the source note.

Across the 44 models with extractable quotes, 82.6% of the 437,886 extracted quotes were verified as present in the corresponding note. At the response level, an unverified quote was less common in responses with a correct binary judgment (21.4% of 120,466 correct responses containing at least one quote) than in incorrect ones (29.3% of 21,318 such incorrect responses), and this direction held for 41 of the 44 models with sufficient correct and incorrect responses to compare (Supplementary Table 12). We therefore find no evidence that models systematically reach correct conclusions via fabricated evidence; if anything, unverifiable quotes are more associated with incorrect answers.

This automated check is a proxy for, not a substitute for, expert evaluation of reasoning quality. It can only confirm whether a quote's exact wording appears in the note. It also cannot reliably distinguish genuine fabrication from legitimate paraphrasing, reformatting (e.g., of units, dates,

or section headers), or model-specific quoting habits, all of which are scored as “unverified” by this method even when the underlying content is faithful to the note. Because these are the only errors this method can make — it has no mechanism for crediting a truly fabricated quote as verified — the unverified-quote rates reported here should be interpreted as an ***upper bound*** on the true rate of fabricated evidence, not a precise estimate.

Table S12: Percentage of responses containing at least one unverified (unmatched) quoted excerpt, overall and split by whether the model's binary judgment matched the reference standard, alongside each model's overall missing/unparseable response rate. Models with a high missing rate have fewer responses contributing to this analysis and their unverified-quote rates should be read with that in mind. Models sorted by overall unverified rate, ascending.

Model	% missing responses	% unverified (overall)	% unverified (correct)	% unverified (incorrect)
Qwen 1 14B	65.0	7.2	9.0	0.0
Qwen 2 VL 2B	0.1	10.0	5.9	33.3
Qwen 1.5 1.8B	0.4	10.2	7.8	10.9
Qwen 2.5 VL 7B	0.0	11.9	11.3	16.3
Qwen 2 1.5B	0.3	12.6	11.2	18.9
Llama 3 8B	2.1	15.8	13.3	21.1
Claude 3.5 Sonnet v1	0.1	16.2	16.1	16.8
Llama 3.3 70B	0.4	16.4	16.2	18.6

Model	% missing responses	% unverified (overall)	% unverified (correct)	% unverified (incorrect)
Qwen 2.5 VL 3B	1.1	16.8	16.7	16.6
Llama 3.1 70B	0.3	17.1	16.0	25.3
Qwen 1 72B	2.5	17.4	17.2	19.6
Qwen 2.5 1.5B	0.0	17.5	16.6	21.7
Qwen 1.5 4B	0.0	17.6	19.1	14.8
Qwen 2.5 14B	0.1	17.7	17.6	18.9
Llama 3.2 VL 90B	0.2	17.8	16.9	23.8
Qwen 2 VL 72B	0.0	17.8	16.5	24.1
Qwen 2.5 VL 72B	0.6	17.9	17.7	19.6
Claude 3.5 Sonnet v2	0.3	18.3	17.7	24.3
Qwen 1.5 110B	0.5	18.5	18.5	18.9
Qwen 2.5 72B	0.1	18.7	18.4	23.2
Llama 3 70B	1.4	18.7	18.1	23.3
Qwen 2.5 7B	0.1	19.2	18.6	25.1
Qwen 2 VL 7B	0.4	19.2	18.6	24.3
Qwen 2.5 32B	0.1	19.4	19.3	21.5

Model	% missing responses	% unverified (overall)	% unverified (correct)	% unverified (incorrect)
GPT-4	0.1	19.5	18.9	22.8
Llama 3.1 405B	0.2	19.8	19.2	25.6
Qwen 2 72B	0.3	20.1	19.2	25.7
Qwen 1.5 72B	0.0	20.1	19.4	24.0
Qwen 1.5 32B	0.5	21.7	20.6	27.1
Qwen 2 7B	1.7	22.7	21.7	25.2
Llama 3.1 8B	0.5	23.3	20.9	35.2
Qwen 1.5 14B	0.3	23.6	22.0	29.1
Qwen 1.5 7B	0.4	25.4	22.5	30.6
Llama 3.2 VL 11B	0.2	26.1	23.5	39.4
Qwen 2.5 3B	0.6	27.3	25.8	36.9
Llama 3.2 3B	0.8	29.0	24.0	45.1
Qwen 2.5 0.5B	0.4	31.5	23.9	36.6
Llama 3.2 1B	1.2	38.4	34.3	41.4
Llama 2 70B	30.8	40.7	37.6	51.8
Llama 2 7B	30.7	43.7	35.6	48.0

Model	% missing responses	% unverified (overall)	% unverified (correct)	% unverified (incorrect)
Qwen 1 7B	1.2	48.1	39.6	54.7
GPT-4o	0.3	53.0*	52.0	63.9
Llama 2 13B	31.0	59.0	59.0	59.2
Qwen 1 1.8B	1.9	64.3	62.5	65.2

**NOTE: GPT-4o's response-level unverified-quote rate (53.0%) is quite high relative to other high-performing models despite a more moderate quote-level rate (27.1%). This largely reflects GPT-4o's substantially higher quoting volume — 3.9 quotes per response, versus 1.6–2.9 among the nine next-best-performing models by macro F_1 — which increases the chance that at least one quote in a given response fails this strict, verbatim-only check. As with the dataset overall, unverified quotes were more common among GPT-4o's incorrect responses (63.9%) than its correct ones (52.0%).*

Agreement between candidate judge models used to parse free-text responses

GPT-o3-mini-high was used to parse every model's free-text response into a binary yes/no answer for the published analysis. To assess whether this parsing step introduces unmeasured errors, we additionally parsed the same free-text responses with three other candidate judge models (GPT-4, GPT-4o, and Claude 3.5 Sonnet) and compared their binary judgments to GPT-o3-mini-high's on the subset of responses both judges in each comparison could parse (Supplementary Table 13). Agreement exceeded 98% and Cohen's kappa exceeded 0.95 for all

three comparisons, in the "almost perfect" agreement range. As a further check, each candidate judge's binary output was compared directly against the reference standard: accuracy against ground truth was nearly identical across all four judges (80.0-81.2%), so GPT-o3-mini-high is not an outlier in either direction. This indicates the parsing step is highly consistent across independent judge models and is unlikely to be a substantial source of error in the reported benchmark metrics. We note this cross-model agreement cannot fully rule out an error shared by all judge models (e.g., a systematically ambiguous note phrasing that misleads every LLM judge in the same way, which only manual review could detect); this as a limitation of the automated parsing approach.

Table S13: Agreement between GPT-o3-mini-high (the judge used for the published analysis) and three alternate candidate judge models, on the free-text-to-binary parsing task, computed on responses both judges in each comparison were able to parse.

Alternate judge model	n (both parseable)	Agreement with GPT-o3-mini-high	Cohen's kappa
GPT-4	485,750	98.2%	0.952
GPT-4o	478,389	99.0%	0.973
Claude 3.5 Sonnet	480,179	99.1%	0.974

Supplementary Figures

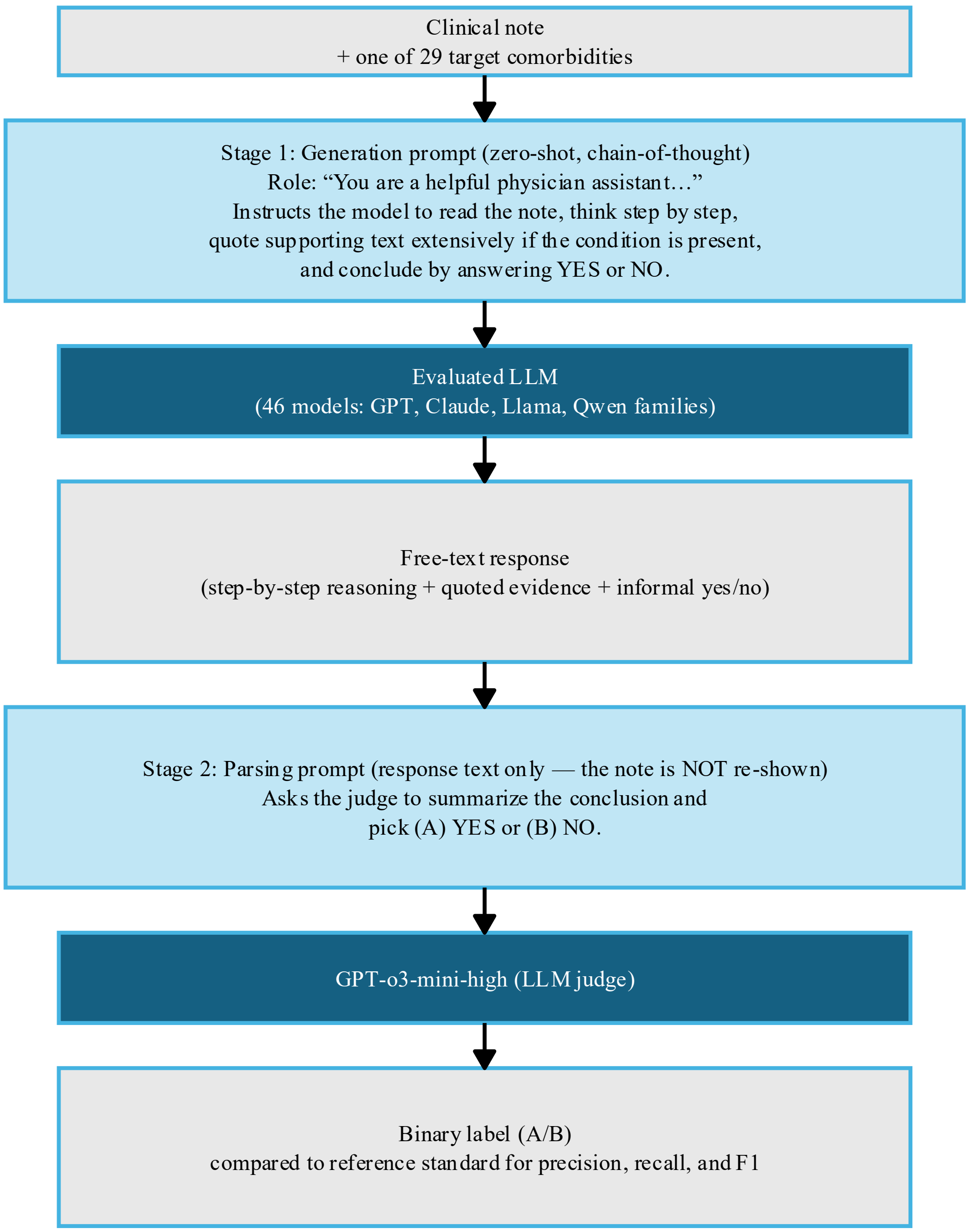

Figure S1: Overview of the prompting strategy used in this study

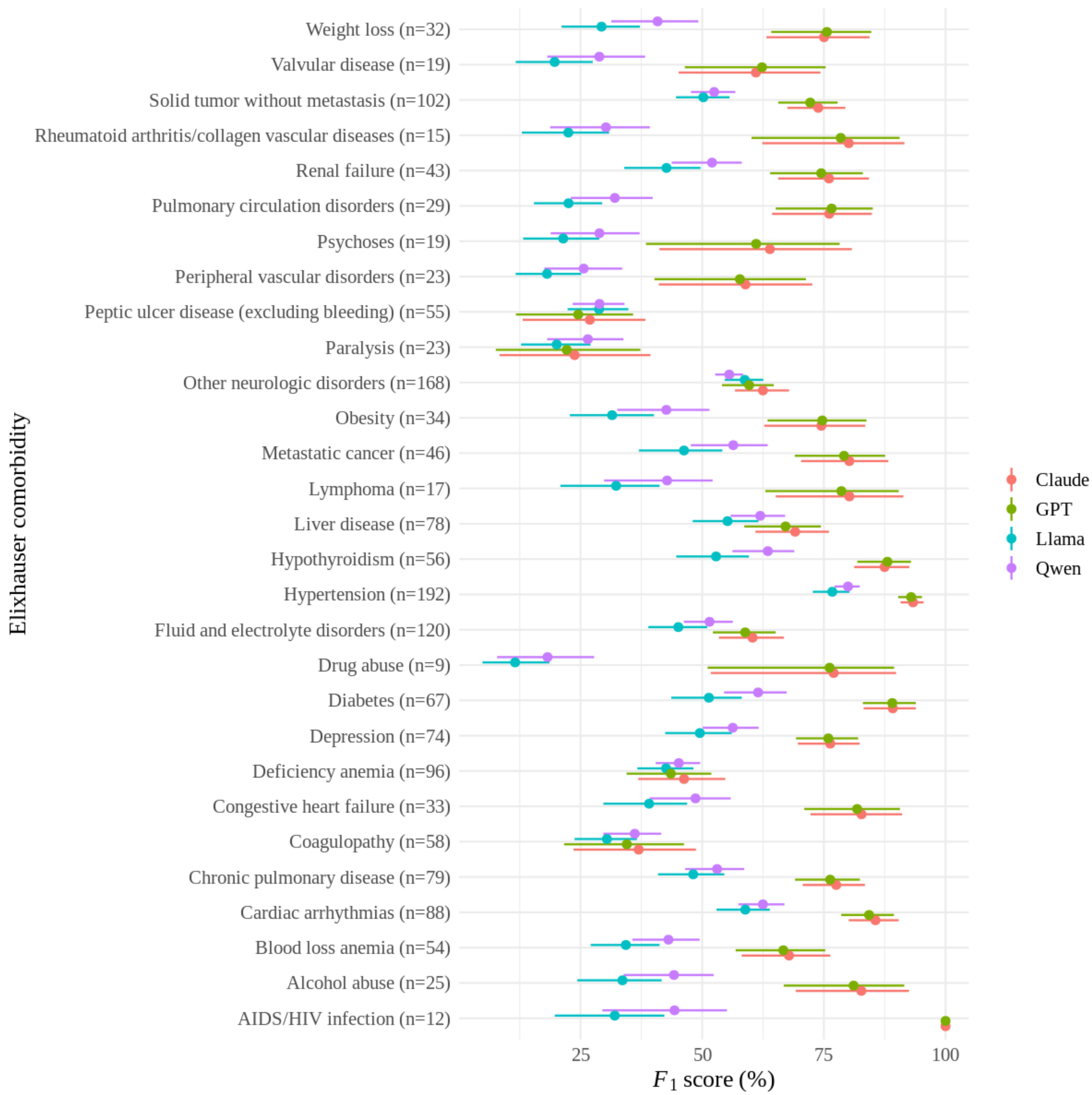

Figure S2:Estimated mean F_1 scores by diagnosis for Claude, GPT, Llama and Qwen models
